# The Effects of Neuregulin on Cardiac Myosin Light Chain Kinase Gene-Ablated Hearts

**DOI:** 10.1371/journal.pone.0066720

**Published:** 2013-06-11

**Authors:** Audrey N. Chang, Jian Huang, Pavan K. Battiprolu, Joseph A. Hill, Kristine E. Kamm, James T. Stull

**Affiliations:** 1 Department of Physiology, University of Texas Southwestern Medical Center, Dallas, Texas, United States of America; 2 Department of Internal Medicine (Cardiology), University of Texas Southwestern Medical Center, Dallas, Texas, United States of America; 3 Department of Molecular Biology, University of Texas Southwestern Medical Center, Dallas, Texas, United States of America; Brigham & Women's Hospital - Harvard Medical School, United States of America

## Abstract

**Background:**

Activation of ErbB2/4 receptor tyrosine kinases in cardiomyocytes by neuregulin treatment is associated with improvement in cardiac function, supporting its use in human patients with heart failure despite the lack of a specific mechanism. Neuregulin infusion in rodents increases cardiac myosin light chain kinase (cMLCK) expression and cardiac myosin regulatory light chain (RLC) phosphorylation which may improve actin-myosin interactions for contraction. We generated a cMLCK knockout mouse to test the hypothesis that cMLCK is necessary for neuregulin-induced improvement in cardiac function by increasing RLC phosphorylation.

**Principal Findings:**

The cMLCK knockout mice have attenuated RLC phosphorylation and decreased cardiac performance measured as fractional shortening. Neuregulin infusion for seven days in wildtype mice increased cardiac cMLCK protein expression and RLC phosphorylation while increasing Akt phosphorylation and decreasing phospholamban phosphorylation. There was no change in fractional shortening. In contrast, neuregulin infusion in cMLCK knockout animals increased cardiac performance in the absence of cMLCK without increasing RLC phosphorylation. In addition, CaMKII signaling appeared to be enhanced in neuregulin-treated knockout mice.

**Conclusions:**

Thus, Neuregulin may improve cardiac performance in the failing heart without increasing cMLCK and RLC phosphorylation by activating other signaling pathways.

## Introduction

Neuregulin is a growth factor which signals to the ErbB family of receptor tyrosine kinases [1]. Cardiac myocytes of the heart express ErbB2/4 receptor tyrosine kinases and neuregulin treatment is associated with several advantageous responses which support its use in human patients with heart failure [2]–[5]. In mouse hearts, treatment with the EGF-like domain of neuregulin can stimulate cardiomyocyte proliferation and improve function after myocardial infarction [2]. However, due to the complexity of growth factor signaling, numerous kinases are reported to be activated by neuregulin, and a specific mechanism for the improvement in cardiac function remains elusive [1], [5], [6]. The reported increase in cardiac myosin light chain kinase (cMLCK) expression with increased cardiac myosin regulatory light chain phosphorylation may be a mechanism through which neuregulin treatment increases cardiac performance [7].

Cardiac myosin exists as a hexamer, each comprised of two heavy chain subunits, each with two types of light chain subunits, an essential light chain and regulatory light chain (RLC). RLC is phosphorylated in cardiac muscle by its dedicated kinase cMLCK at a specific serine in the N-terminus [8]–[10]. This phosphorylation increases the Ca^2+^ sensitivity of myofilament contraction and maximal force development [11]–[13]. Molecular mechanisms involve increased rate of myosin attachment to actin thin filaments and slowing of the crossbridge power stroke due to increased stiffness of the myosin lever arm [14]. Thus, myosin phosphorylation cooperatively sustains thin filament activation with prolongation of the relaxation cycle.

RLC is phosphorylated in normal beating hearts. The extent of RLC phosphorylation is about 0.4 mol phosphate per mol RLC in different animal species, and this basal phosphorylation is sufficient to enhance contractile force in permeable fibers [11], [12]. The physiological importance of cardiac RLC phosphorylation is supported by numerous mouse models. Decreased RLC phosphorylation in the heart by overexpression of a non-phosphorylatable mutant RLC or a knock-in mutation of the phosphorylation site have shown that this post-translational modification is necessary for optimal cardiac function [14]–[16]. Additionally, increased cardiac RLC phosphorylation by kinase overexpression in transgenic mice inhibited stress-induced responses [17], [18].

Although the physiological importance of RLC phosphorylation is demonstrated with genetically modified mice, determinants for cMLCK activity have yet to be identified. There is agreement that the kinase has a low specific activity [8], [9] which is reflected in the much lower MLCK activity in cardiac muscle homogenates compared to those for skeletal or smooth muscles [11]. Skeletal and smooth muscle MLCKs are activated by the binding of Ca^2+^/calmodulin to homologous calmodulin binding sequences to displace an autoinhibitory segment from the catalytic cleft [19]. However, the Ca^2+^/calmodulin-dependency of cMLCK activity is controversial even though this kinase has a calmodulin binding sequence and autoinhibitory segment similar to the skeletal and smooth muscle MLCKs [8], [9]. It has also been suggested that cMLCK may be phosphorylated, thus implying post-translational regulation of activity, but no details are specifically known at this time.

Ablation of cMLCK in a hypomorphic mouse model previously described by our laboratory attenuated RLC phosphorylation [10]. Furthermore, cardiac performance was depressed. The hearts were slightly enlarged showing fibrosis in older mice [10], similar to previous observations in which a knock-in mutation in RLC prevented phosphorylation [14]. We have extended these studies by generating a conventional knockout model where the *neo* cassette, floxed gene, and Cre recombinase transgene were removed. This knockout mouse line has no cMLCK, and shows attenuated RLC phosphorylation and diminished cardiac function in young mice, similar to previously described results for the hypomorph [10]. However, diminished cardiac performance occurred with modest hypertrophy and no fibrosis, supporting a direct causative effect on cardiac function that is not secondary to changes in tissue morphology. We therefore sought to determine in these mice whether cMLCK and RLC phosphorylation were necessary for improvement of cardiac function by neuregulin. Surprisingly, we found neuregulin treatment restored cardiac performance in the absence of RLC phosphorylation *in vivo,* and have investigated potential alternative mechanisms.

## Materials and Methods

### Animals

All procedures were performed in accordance with the Institutional Animal Care and Use Guidelines. The protocol was approved by the University of Texas Southwestern Medical Center Institutional Animal Care and Use Committee (APN#0166-06-11-1). Animals were housed under specific pathogen free conditions in our AAALAC certified rodent facilities; surgeries were performed under isoflurane anesthesia and mice were monitored during recovery.

A conventional knockout of cMLCK was generated from the floxed mice previously described [10]. Mice with floxed allele were bred with the CAG-Cre transgenic line which allowed for excision of the floxed allele irrespective of transmission of the CAG-Cre gene [20]. Heterozygous animals which had the knockout allele, *Mylk3* ^+/−^, but not the CAG-Cre gene, were selected for subsequent breeding. Littermate wildtype mice were used as controls. For all studies, 12–15 week old male mice were used.

### RNA Analysis

Total RNA was purified from isolated heart ventricles with TRIzol reagent (Invitrogen) according to the manufacturer's instructions. High Capacity cDNA Reverse Transcription Kit (Applied Biosystems) was used to synthesize cDNA from 0.5 microgram of RNA. Quantitative PCR was performed using the following TaqMan® probes purchased from Applied Biosystems: ANP, Mm01255748_g1; BNP, Mm01255770_g1; Col1a2, Mm00483888_m1; Myh6, Mm00440359_m1; Myh7, Mm00600555_m1; and 18S, 4333760F. Analyses were performed by the comparative CT method. Initial data were normalized to 18S; relative values were obtained by normalizing to the median for *Mylk3* ^+/+^.

### Echocardiography

Echocardiograms were performed on conscious, gently restrained mice using either a Sonos 5500 system with a 15-MHz linear probe or Vevo 2100 system with a MS400C scanhead. Left ventricular internal diameter at end-diastole (LVIDd) and end-systole (LVISd) were measured from M-mode recordings. Percent fractional shortening was calculated as ((LVIDd − LVISd)/LVIDd)x100. Measurements of interventricular septum thickness, left ventricular internal diameter, and left ventricular posterior wall thickness were made from two-dimensional parasternal short axis views in diastole [21]. All measurements were made at the level of papillary muscles.

### Animal Protocols

Mice were treated with 0.2% BSA/PBS or neuregulin (EFG-like domain, amino acids 176–246, R&D Systems) 5ug/kg/h in 0.2% BSA/PBS for 7 days through surgically implanted Alzet® mini-osmotic pump (model 2001, Durect Corp.) [7]. All animals were anesthetized with isoflurane during the surgical procedure and treated with analgesics to minimize post-operative pain. Echocardiographic measurements were performed before and after the 7 day treatment. At the end of the treatment, mice were anesthetized (250 mg/kg Avertin, intraperitoneal) and quickly weighed. Whole hearts were immediately removed, weighed, and ventricles frozen in liquid nitrogen. All tissue collections were performed in the afternoon between 3–5:00 PM. Ventricles to be used for phospholamban phosphorylation measurements were snap frozen with clamps pre-chilled in liquid nitrogen immediately after dissection in less than 30 seconds. Tibial length was measured with a micro-caliper.

### Statistical Analyses

Data are expressed as mean ± S.E. Statistical evaluation was carried out in GraphPad Prism using an unpaired Student's t-test for two comparisons or paired t-test for comparison of same sample before and after treatment. Analysis of variance and Newman-Keuls post-test were used for multiple comparisons. Significance was accepted at a value of p<0.05.

### Immunoblots and Antibodies

Frozen ventricles were ground in liquid nitrogen and an aliquot thawed in 10% trichloroacetic acid containing 10 mM dithiothreitol. Precipitated protein was washed free of acid with three 5-minute washes in ethyl ether and resuspended by vigorous agitation in urea sample buffer (8 M Urea, 20 mM Tris base, 23 mM glycine, 0.2 mM EDTA, 10 mM dithiothreitol) using an orbital shaker (IKA Vibrax VXR) set at 1400 rpm for 30 minutes at room temperature. Complete denaturation and solubilization was achieved by addition of urea crystals and prolonged agitation. Protein samples were subjected to centrifugation at 10,000 x g for 2 minutes and protein concentrations in supernatant fractions measured by Bradford assay. Proteins (2 – 40 µg) were subjected to SDS-PAGE after boiling in Laemmli buffer, transferred to PVDF (Immobilon-P, Millipore) or nitrocellulose (Protran, Whatman), and blotted by standard procedures. The amount of protein loaded was optimized empirically for each antibody to ensure density measurements were proportional to the amount of protein.

Measurements of RLC phosphorylation in heart homogenates were performed by urea/glycerol-PAGE and immunoblotting, as previously described [22]. The urea/glycerol-PAGE system separates phosphorylated RLC from non-phosphorylated RLC, allowing a direct quantitative measure of RLC phosphorylation in terms of fraction phosphorylated RLC of total RLC. As the separation results from a single phosphate, data may also be calculated as mol of phosphate/mol RLC. Briefly, polyacrylamide gels containing 40% glycerol were pre-electrophoresed for 1 h at 400 V at room temperature in a mini-gel apparatus. Reservoir buffer contained 20 mM Tris base and 23 mM glycine, pH 8.6; thioglycolate and dithiothreitol (2.3 mM each) were included in the upper reservoir. Samples (2 µg in urea sample buffer) were subjected to electrophoresis for 90 minutes at 400 V at room temperature, then transferred to a PVDF membrane for 1 hour at 0.3 A at 4°C. Post-transfer, proteins were fixed onto the PVDF membrane with 0.4% glutaraldehyde/PBS for 15 minutes at room temperature. The membrane was then rinsed 3x in PBS and immunoblotted with antibody to cardiac RLC.

Antibodies specific to cardiac RLC and cMLCK were reported previously [10], [17]. Antibody to phospho-phospholamban, phosphorylated PLB_Thr17 was purchased from Badrilla Ltd. Antibodies to extracellular signal-regulated protein kinase, ERK (4695); phosphorylated ERK, pERK (43770); phosphorylated cardiac troponin I, pTnI (4004), AKT (4685); pAkt_Ser473 (4060) were purchased from Cell Signaling Technology. Antibodies to phosphorylated focal adhesion kinase, pFAK_Tyr397 (44-624G) and pFAK_Tyr861 (44-626G) were purchased from Invitrogen Corporation. Antibodies to total FAK (sc-558) and GAPDH (25778) were purchased from Santa Cruz Biotechnology Inc. Antibodies to total cardiac TnI (MAB1691), total PLB (05-205), and phosphorylated PLB_Ser16 (07-052) were purchased from Millipore. Antibodies to pCaMKII_Thr286 (MA1-047); sarcoplasmic reticulum Ca^2+^ transport ATPase, SERCA2a (MA3-919); total protein kinase D, PKD (2052P); and pPKD_Ser916 (2051P) were purchased from Pierce. Antibody to phosphorylated cardiac myosin binding protein C, pMybp-C_Ser282 (ALX 215-057), was purchased from Enzo Life. Antibody to total CaMKIIδ was a generous gift from Donald Bers (University of California, Davis).

## Results

### Lack of cMLCK and RLC phosphorylation did not lead to significant hypertrophy in adult knockout mice

**Figure 1:**
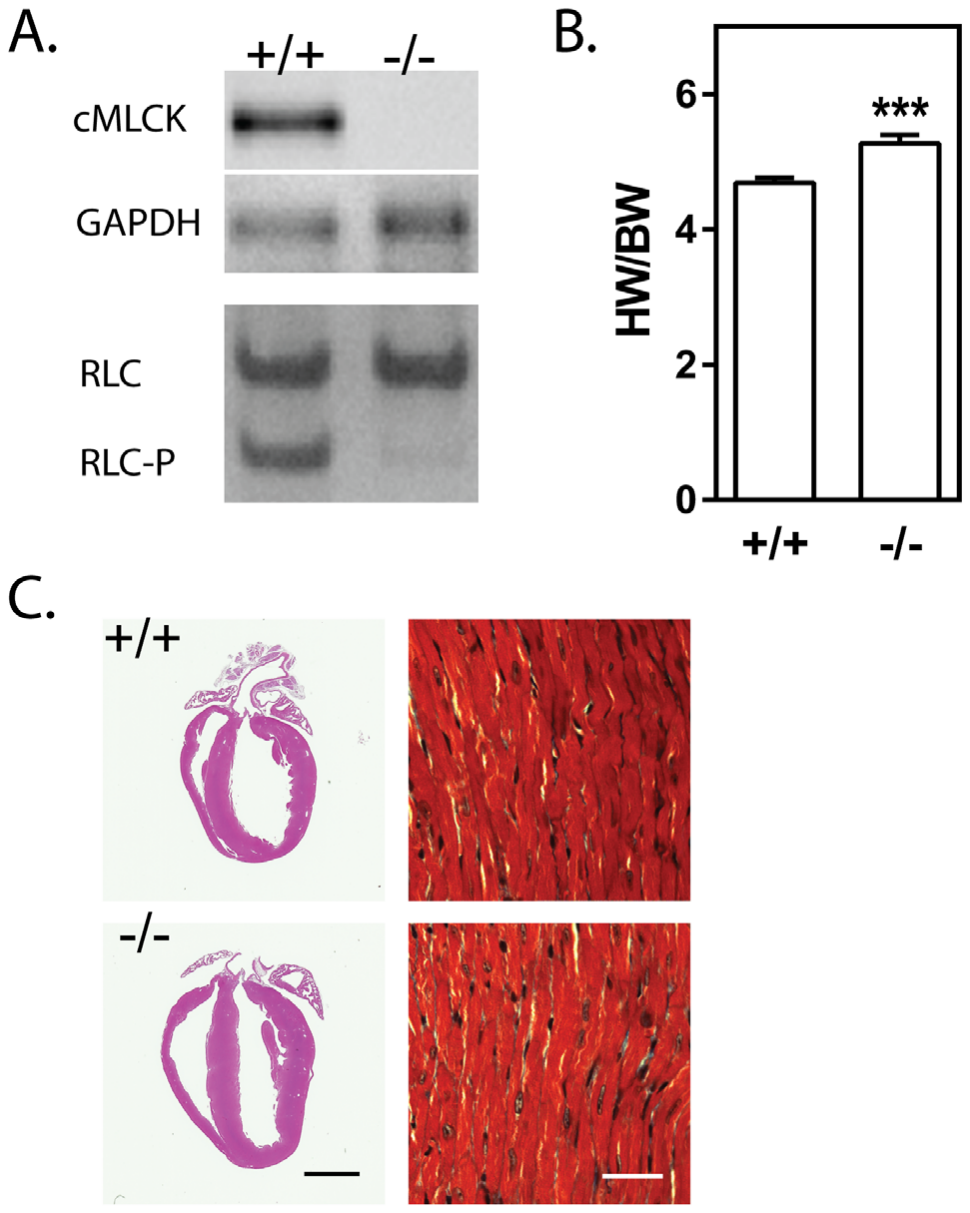
Characterization of hearts from cMLCK knockout mice. A) Representative image of immunoblot analysis of wild-type (+/+) and cMLCK knockout (−/−) hearts for cMLCK protein (upper panel) and phosphorylation of cardiac regulatory light chain (nonphosphorylated cRLC; monophosphorylated cRLC-P). GAPDH is shown as loading control. B) Ratio of mouse heart weight to body weight. *** P<0.001 Student’s t-test, one tailed. C) Representative image of hematoxylin and eosin stain of fixed 4-chamber view of 3 month old male wildtype and cMLCK knockout mouse hearts. Trichrome-stained and magnified area of left ventricular free wall is shown; inset black bar-2 mm, white bar-200 µm.

A mouse line of cMLCK knockout animals was generated by breeding cMLCK floxed mice [10] with CAG-Cre transgenic mice [20]. Mice with the knockout alleles but negative for the Cre recombinase transgene, were selected for breeding of homozygous knockout and wildtype littermate control mice. Hearts from knockout mice lacked cMLCK protein and had attenuated cardiac RLC phosphorylation (Fig 1A) as previously reported [10], [14]. Heart weight/body weight ratios were increased significantly from 4.69±0.08 to 5.27±0.12 in 10–15 week old males (Fig 1B). Mild dilation was visible by histology with no notable fibrosis or myofibrillar disarray (Fig 1C). Lack of a more robust hypertrophy with cMLCK ablation was consistent with the absence of significant differences in the amounts of mRNA for genes known to be up regulated in hypertrophy (Table 1).

**Table 1:** Comparison of hypertrophy-associated mRNA.

	+/+	−/−
ANP	0.98±0.11	1.24±0.07
BNP	1.00±0.01	0.78±0.11
Col1a2	0.95±0.18	1.06±0.05
Myh6	1.02±0.10	1.08±0.05
Myh7	1.22±0.37	1.57±0.17

Relative quantities normalized to the mean wildtype are shown. Abbreviations are: ANP, natriuretic peptide A; BNP, natriuretic peptide B; Col1a2, collagen type 1 alpha 2; Myh6, cardiac alpha myosin heavy chain; Myh7, cardiac beta myosin heavy chain. Significant differences were analyzed by Student’s t test, two-tailed, GraphPad software. N = 3 for wildtype mice (+/+), 6 for cMLCK knockout mice (−/−). There were no significant differences.

### Neuregulin infusion improved cardiac function of cMLCK knockout animals

**Figure 2:**
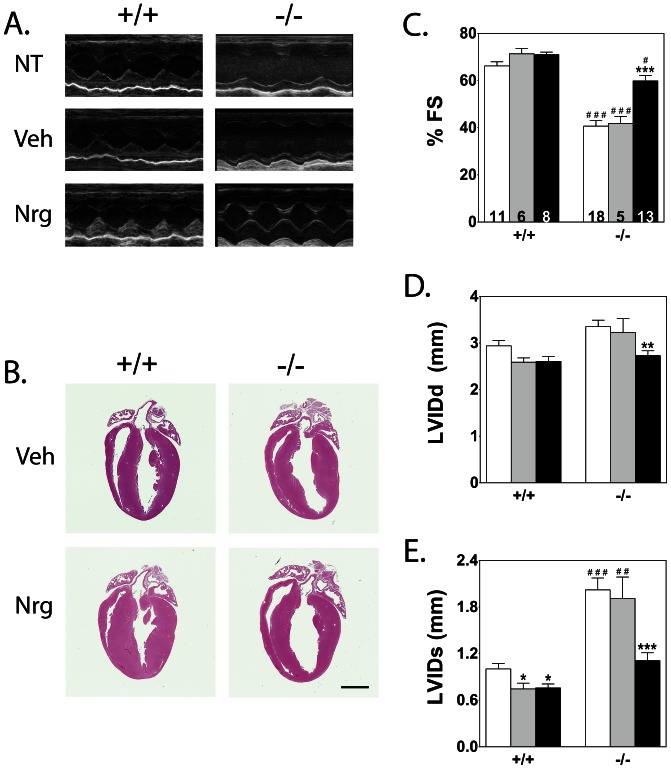
Effects of vehicle and neuregulin treatment on heart morphology and function . A) Representative echocardiography loop images of non-treated (NT), vehicle-treated (Veh), and neuregulin-treated (Nrg) wildtype (+/+) and cMLCK knockout (−/−) hearts. B) Representative image of hematoxylin and eosin stain of fixed 4-chamber view of 3 month old male wildtype and cMLCK knockout, Veh and Nrg mouse hearts. Inset scale bar-2 mm. C, D, E) Percent fractional shortening (%FS), and left ventricular internal dimension at diastole (LVIDd) and systole (LVIDs). Sample number for each treatment group is indicated within each %FS bar. Non-treated shown in white, vehicle in gray, and neuregulin treatment in black. Significance was determined by 1-way ANOVA and Newman-Keuls multiple comparison test using GraphPad software. * P<0.05, ** P<0.01, *** P<0.001 compared with NT within its genotype group;^ #^ P<0.05, ^##^ P<0.01, ^###^ P<0.001 compared with treatment-matched wildtype group.

In order to ascertain whether cMLCK is necessary for neuregulin-associated increase in RLC phosphorylation and thereby improved cardiac function, we infused neuregulin into cMLCK knockout mice for seven days [7]. Representative echocardiographic measurements showed poor cardiac performance in non-treated and vehicle-treated knockout mice, which is significantly improved with neuregulin treatment (Fig 2A). Histological analyses suggested modest hypertrophy in the wildtype group with neuregulin treatment (Fig 2B) and up-regulation of hypertrophy markers measured by QPCR (data not shown). However, there were no apparent changes in cardiac performance between vehicle and neuregulin-treated wildtype mice (Fig. 2 C, D, and E). Quantitation of cardiac function showed low fractional shortening in cMLCK knockout animals that was improved with neuregulin treatment to values approaching those obtained with wildtype mice (Fig 2C). Left ventricular internal diastolic dimension improved, but the systolic dimension improved dramatically with neuregulin treatment (Fig 2D, E).

### Neuregulin infusion caused ventricular hypertrophy

Comparison of the interventricular-septal and posterior wall thickness at diastole before and after treatment shows significant increases in thickness associated with neuregulin treatment for hearts from wildtype or cMLCK knockout mice (Table 2). While a small but significant increase in heart weight to body weight ratios were seen between non-treated wildtype and cMLCK knockout groups, the difference did not extend to ventricular wall thickness measurements. Furthermore, while wall thickness increased significantly after neuregulin treatment, there were no significant differences between genotype groups. Thus, hypertrophy responses were modest relative to changes observed in cardiac performance in neuregulin-treated cMLCK knockout mice.

**Table 2:** Morphometric and echocardiographic parameters.

	BW (g)	HW (mg)	TL (mm)	HW/TL	HW/BW	HR (bpm)	IVSd (mm)	PWd (mm)
+/+ NT	24.8±1.1	116±5	18.0±0.1	6.48±0.23	4.69±0.08	694±13	0.77±0.05	0.70±0.04
+/+ Veh	28.5±2.7	139±6	18.3±0.2	7.62±0.33	4.94±0.26	728±10	0.71±0.10	0.67±0.06
+/+ Nrg	26.6±1.2	134±7	17.9±0.2	7.47±0.31	5.04±0.08	649±12 ^δ^	0.94±0.07 ^δ^	0.96±0.08 ^δ^
−/− NT	24.3±0.8	128±3	17.9±0.1	7.13±0.14	5.27±0.12 ^#^	646±25	0.74±0.02	0.74±0.03
−/− Veh	25.1±1.1	132±6	17.8±0.2	7.39±0.28	5.27±0.13 ^#^	690±23	0.80±0.07	0.83±0.07
−/− Nrg	23.8±0.5	128±3	17.7±0.1	7.22±0.15	5.38±0.05 ^#^	691±15	0.85±0.04^ δ^	0.99±0.04 ^δ^

N = 4 or more. Abbreviations are: BW, body weight; HW, heart weight; TL, tibial length; HR, heart rate; IVSd, interventricular septal wall diameter; PWd, posterior wall diameter. Significance determined using GraphPad software. ^#^P<0.05 compared with non-treated wildtype (+/+ NT) hearts by 1 way ANOVA, Newman-Keuls multiple comparisons test; ^δ^P<0.05 compared with pre-treatment values (not shown) by paired t-test, parametric, one-tailed.

### pAkt was increased with neuregulin infusion in wildtype

**Figure 3:**
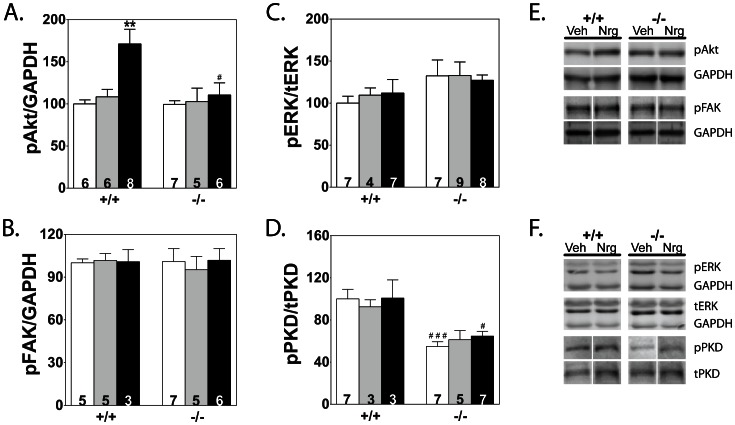
Effects of vehicle and neuregulin treatment on selected kinases . A–D) Quantitation of phosphorylated proteins compared with respective total protein or GAPDH for each treatment group is shown. Sample number is indicated within each bar. Significance was determined by 1-way ANOVA and Newman-Keuls multiple comparison test using GraphPad software. ** P<0.01 compared with NT within its genotype group; ^#^ P<0.05, ^###^ P<0.001 compared with treatment-matched wildtype group. E, F) Representative immunoblot images for each protein are shown for Veh and Nrg treated wildtype (+/+) and cMLCK knockout mice (−/−).

There are several reports of protein kinases being upregulated with neuregulin treatment [7], [23], [24]. Immunoblot analyses of phosphorylated and total kinase amounts showed only pAkt at Ser473 was increased with neuregulin treatment in wildtype animals (Fig 3A). There were no significant differences between the treatment groups in FAK or ERK phosphorylation or amounts (Fig 3B, C). Less phosphorylated PKD was present in knockout animals, but the amounts were not altered with vehicle or neuregulin treatment (Fig 3D). Representative blots for each protein are shown (Fig 3E, F).

### Neuregulin treatment did not increase phosphorylation of myofilament-associated calcium sensitive proteins

**Figure 4:**
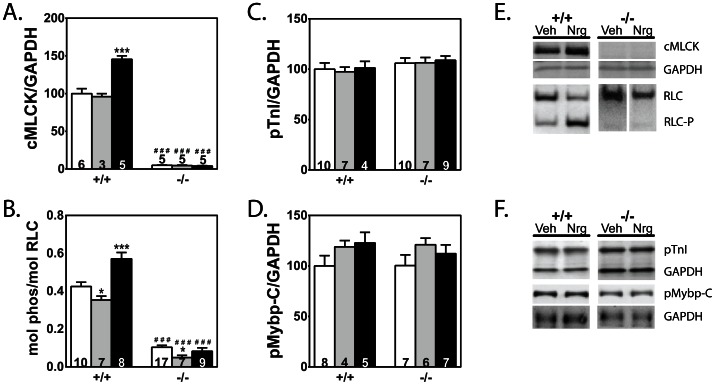
Effects of vehicle and neuregulin treatment on phosphorylation of selected myofilament-associated proteins . Values for hearts from non-treated (white bars), vehicle-treated (gray bars), and neuregulin-treated (black bars) animals are shown. A) Quantitation of cMLCK amounts compared to GAPDH for each treatment group is shown. B–D) Quantitation of phosphorylated proteins compared with respective total protein or GAPDH for each treatment group is shown. Sample number is indicated within each bar. Significance was determined by 1-way ANOVA and Newman-Keuls multiple comparison test using GraphPad software. *P<0.05, ***P<0.001 compared with NT within its genotype group; ^###^ P<0.001 compared with corresponding wildtype group. E, F) Representative immunoblot images for each protein are shown for Veh and Nrg treated wildtype (+/+) and cMLCK knockout (−/−).

To test possible associations of neuregulin with regulation of sarcomeric proteins that increase myocardial contractility, sarcomeric protein phosphorylation was measured. Total cMLCK and RLC phosphorylation both increased in wildtype neuregulin-treated hearts, but not in the cMLCK knockout group (Fig 4A, B, E). The content of cMLCK increased 52% while RLC phosphorylation increased by 62% in hearts from wildtype animals. In contrast, total protein expression and phosphorylation of contractile proteins troponin I (TnI) and myosin-binding protein C (Mybp-C) did not change in any of the groups (Fig 4C, D, F).

### CaMKIIδ amounts are increased in cMLCK knockout neuregulin-treated mice

**Figure 5:**
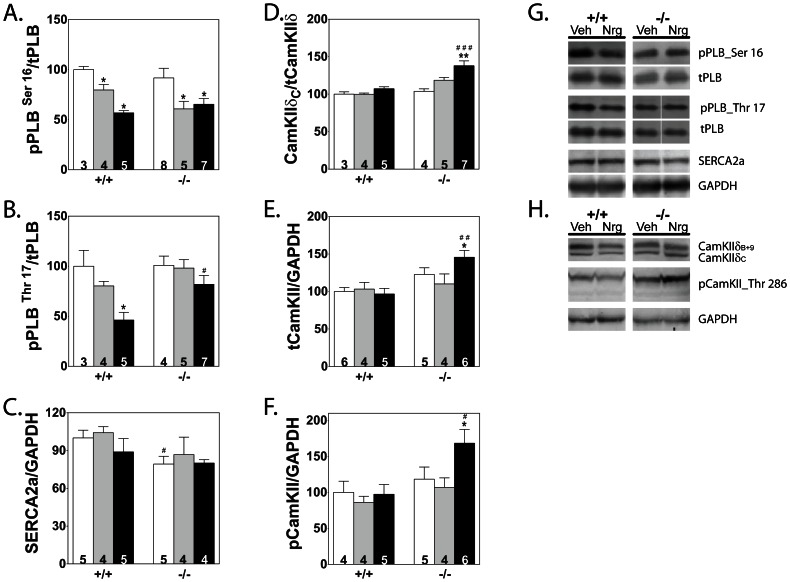
Effects of vehicle and neuregulin treatment on calcium handling proteins not associated with the myofilament . A–F) Quantitation of proteins compared with respective total protein or GAPDH for each treatment group is shown. Values for hearts from non-treated (white bars), vehicle-treated (gray bars), and neuregulin-treated (black bars) animals are shown. Sample number is indicated within each bar. Significance was determined by 1-way ANOVA and Newman-Keuls multiple comparison test using GraphPad software. *P<0.05, **P<0.01 compared with NT within its genotype group; ^#^ P<0.05,^ ##^ P<0.01, ^###^ P<0.001 compared with treatment-matched wildtype group. G, H) Representative immunoblot images for each protein are shown for Veh and Nrg treated wildtype (+/+) and cMLCK knockout (−/−).

Cardiac performance may also be affected by changes in Ca^2+^ handling by the sarcoplasmic reticulum via the Ca^2+^-ATPase pump (SERCA2a) and its regulator, phospholamban (PLB). Measurement of phosphorylated PLB showed a decrease in both the cyclic-AMP and CaMKII-regulated sites, Ser16 and Thr17, in neuregulin-treated hearts from wildtype mice (Fig 5A, B, G). However, in cMLCK knockout neuregulin group, the amount of phosphorylated PLB Thr17 did not decrease and amounts were significantly higher in neuregulin-treated hearts from cMLCK knockout mice compared to phosphorylated PLB in hearts from wildtype animals. In addition, there was a small decrease in the amount of total SERCA2a in the cMLCK knockout group, but no difference was observed between vehicle and neuregulin treatment groups (Fig 5C, F). The observed differences in phosphorylated PLB Thr17 between wildtype neuregulin-treated and cMLCK knockout neuregulin-treated hearts were extended with measurements of CaMKIIδ_C_ (cytoplasmic CaMKIIδ), the kinase which phosphorylates that residue. Total CaMKIIδ (sum of the cytoplasmic and nuclear CaMKIIδ isoforms, C and B+9, respectively), CaMKIIδ_C_ and phosphorylated CaMKII were all increased only in hearts from cMLCK knockout mice after treatment with neuregulin (Fig 5D–F, H).

## Discussion

We previously reported a hypertrophic phenotype associated with significant fibrosis and decreased cardiac performance in cMLCK hypomorphic knockout mice generated by insertion of the neo cassette into the *Mylk3* gene [10]. A similar hypertrophy was noted with a knockin mutation that prevented RLC phosphorylation but with milder fibrosis [14]. The current conventional cMLCK knockout mice had a milder phenotype with modest hypertrophy and no fibrosis, similar to recently published results [18]. We noted that the percent fractional shortening previously decreased 37% [10] while there was a 23% decrease with the conventional cMLCK knockout mice. Therefore, we used these conventional knockout mice in the absence of complicating pathological changes to determine if RLC phosphorylation was necessary for neuregulin signaling to improve cardiac performance.

Neuregulin treatment resulted in an increase in cMLCK expression and RLC phosphorylation in hearts from wildtype mice similar to results reported for rats [7]. In contrast to results obtained with hearts from wildtype animals, neuregulin treatment increased fractional shortening in impaired hearts from cMLCK knockout mice. Thus, improved cardiac function in failing heart is not dependent on RLC phosphorylation. These results were surprising considering the critical role of RLC phosphorylation in cardiac regulation of actin-myosin interactions [11], [14], [18]. The apparent impairment of myofilament function induced by RLC dephosphorylation may be compensated by other signaling mechanisms.

Interestingly a recent publication reported that a 21-fold overexpression of cMLCK increased RLC phosphorylation from 0.34 mol phosphate per mol RLC to only 0.50 mol phosphate per mol RLC in the transgenic hearts, a 32% increase [18]. We found a 52% increase in cMLCK expression increased RLC phosphorylation by 62% in neuregulin versus vehicle-treated hearts from wildtype mice. Thus, neuregulin treatment may be activating cMLCK, potentially by phosphorylation [8]. If another kinase could phosphorylate RLC directly, it is not activated by neuregulin since there was no increase in RLC phosphorylation cMLCK in hearts from knockout mice.

In contrast to reported increases in phosphorylation of different protein kinases [7], [23], [24], we found neuregulin treatment only increased Akt Ser473 phosphorylation in wildtype mice. Activation of Akt is associated with myocyte growth and inhibition of autophagy [25], [26], consistent with the increase in ventricular wall thickness after neuregulin treatment (Table 2). Contrary to comparative increases in the ventricular wall thickness of knockout mice, Akt phosphorylation was not increased. Thus, although Akt is activated by neuregulin, the improvement in cardiac performance in the knockout animals in response to neuregulin may not be dependent on Akt signaling.

While sarcomeric proteins TnI and MyBP-C were not significantly phosphorylated in response to neuregulin, PLB phosphorylation at both the CaMKII and PKA–dependent sites were decreased with neuregulin treatment in wildtype mice. The increase in RLC phosphorylation is predicted to enhance Ca^2+^-dependent actin-myosin interaction. However, the decrease in PLB phosphorylation may counterbalance this response by decreasing Ca^2+^-delivery and thereby attenuating any improvement of cardiac performance associated with increased RLC phosphorylation. The decrease in PLB phosphorylation may be secondary to the decrease in heart rate [27]. The observed decline in heart rate may decrease Ca^2+^ signaling responses related to CaMKII where the amounts of total and phosphorylated CaMKII were not altered in the wildtype hearts after neuregulin treatment, but PLB phosphorylation decreased [27]–[29]. The sensitivity of PLB phosphorylation to changes in heart rate is inferred by the lack of changes in phosphorylation of TnI or MyBP-C.

Comparison of the PKA-dependent and CaMKII-dependent phosphorylation sites in PLB showed that in the cMLCK knockout hearts, only the CaMKII-dependent site was modestly decreased in neuregulin-treated when compared with vehicle-treated hearts. However, the amount of phosphorylated PLB Thr17 in the neuregulin-treated cMLCK knockout hearts was significantly greater than in the neuregulin-treated wildtype hearts. This increase is consistent with CaMKIIδ measurements, where only in the cMLCK knockout, neuregulin-treated group, total and pCaMKII levels were increased. Although the antibody to phosphorylated CaMKII is not specific to CaMKIIδ, parallel signal increases in the neuregulin-treated group supports its activation. Measurement of the cytoplasmic isoform of CaMKIIδ (CaMKIIδ_C_) showed its ratio over total is significantly increased only in the cMLCK knockout, neuregulin-treated group. Overexpression studies of CaMKIIδ isoforms in the heart associates increased CaMKIIδ_C_ levels with poor cardiac function [28], [30], [31], so why CaMKIIδ levels are increased with neuregulin treatment and improved cardiac function in knockout animals remains unclear.

There are numerous differences between rodents and human hearts which must be considered when mouse models are used to study human cardiac diseases. The studies reported here used an engineered heart failure model caused by the ablation of cMLCK and thereby phosphorylated RLC, not yet described in the human population. This model was used to address the specific question of whether neuregulin-induced improvement of cardiac performance was dependent on cMLCK. In summary, heart failure may be improved by neuregulin treatment without cMLCK and increased RLC phosphorylation. The neuregulin-ErbB signaling pathways that improve cardiac function independent of RLC phosphorylation need to be identified.
